# Challenges and outcomes in pediatric and adult kidney transplantation

**DOI:** 10.3389/fped.2026.1779651

**Published:** 2026-04-28

**Authors:** Viola Weeda, Nikolaos Koliakos, Thomas Caës, Adrian Khelif, Dimitri Mikhalski, Pierre Lingier

**Affiliations:** 1 Department of Pediatric Surgery, Queen Fabiola Children’s University Hospital, HUDERF, Hôpital Universitaire de Bruxelles, Université Libre de Bruxelles, Brussels, Belgium; 2 Department of Abdominal Surgery, Erasme, Hôpital Universitaire de Bruxelles, Université Libre de Bruxelles, Brussels, Belgium; 3 Department of Urology, Erasme, Hôpital Universitaire de Bruxelles, Université Libre de Bruxelles, Bruxelles, Belgium

**Keywords:** kidney transplantation, pediatric transplantation, postoperative management, preoperative evaluation, surgical complications

## Abstract

The preoperative evaluation and postoperative management of kidney transplantation are fundamentally tailored to the distinct needs of adult and pediatric populations. The pediatric assessment prioritizes growth, congenital anomalies (like CAKUT), neurodevelopmental progress, and achieving pre-emptive transplantation. In contrast, adult evaluation focuses on comprehensive cardiovascular screening, malignancy risk, and managing accumulated comorbidities, with a growing emphasis on frailty assessment. Surgical technique in children is adapted for size, requiring meticulous microvascular anastomosis and specific urinary reconstruction strategies, especially in the context of complex urological histories. Postoperatively, complication profiles differ adults face higher rates of delayed graft function and cardio metabolic sequelae, while children's unique vascular technical risks and the critical challenge of adolescent non-adherence prevail in children. For both groups of patients, urinary tract infections are a major concern, necessitating structured prophylaxis and management. Looking ahead, pediatric transplantation is exploring minimally invasive robotic techniques. Ultimately, successful long-term outcomes depend on a multidisciplinary, life-stage-specific approach that balances immunological risks with the distinct developmental or chronic disease trajectories of each patient to optimize graft survival and quality of life.

## Highlights

- Renal transplantation is precision medicine in the pre-, per- and postoperative trajectory for both pediatric and adult patients
- Impeccable surgical technique tailored to patient-specific needs should be combined with detail-oriented donor selection whenever possible
- In children, are management of congenital anomalies, growth, and treatment compliance towards adolescence are key priorities during these complex trajectories while in adults, containment of hypertension, diabetes and cardiosclerosis are essential
- Currently, no viable alternative to renal transplantation is at hand, however promising techniques are developed by collaborating bioengineers

## Introduction

Kidney transplantation stands as the optimal treatment for end-stage renal disease, offering superior survival, quality of life, and cost-effectiveness compared to long-term dialysis. This life-saving procedure, however, is a continuum of complex, interdependent phases—from meticulous candidate evaluation and precise surgical implantation to vigilant long-term management (1). Each stage presents unique challenges and critical decisions that directly impact graft and patient outcomes. The success of a transplant is not determined by a single intervention but by the integration of tailored medical, surgical, and psychosocial strategies across the entire patient journey, from the preoperative clinic to lifelong follow-up (2).

Given the profound differences in physiology, pathology, and life-course priorities between pediatric and adult recipients, a unified approach is insufficient. The purpose of this descriptive review is to provide a comprehensive, comparative analysis of kidney transplantation across these two distinct populations. We will examine the divergent paradigms in preoperative assessment, surgical technique, postoperative complication management, and long-term follow up. We will also provide a state-of-the-art overview of novel techniques emerging from the field of bioengineering, being developed with the goal of replacing surgical transplantation in the future. By synthesizing current evidence and guidelines, this review aims to elucidate the specialized principles that guide clinical practice for each group, highlighting how precision medicine and age-adapted strategies are fundamental to optimizing transplantation outcomes for all patients and how they may be successfully applied.

## Methods

A comprehensive narrative literature review was conducted to examine the preoperative evaluation, surgical technique including recent innovation, postoperative management and long-term complications of kidney transplantation from both pediatric and adult perspectives, as well as the progress made thus far by the field of bioengineering in renal replacement therapy development. Searches were performed using the PubMed/MEDLINE, Google Scholar and SCOPUS databases for articles published in the English language between January 2000 and December 2025. Where applicable, pertinent cross-references of previous dates were also perused. The search strategy employed a combination of Medical Subject Headings (Mesh) terms and keywords, including: “Kidney transplantation”; “renal transplantation”; “pediatric”; “children”; “adult”; “elderly”; “preoperative care”; “preoperative evaluation”; “patient selection”; “growth”; “development”; “CAKUT”; “cardiovascular diseases”; “frailty”; “malignancy”; “vascular imaging”; “surgical technique”; “anastomosis”; “urological”; “ureter”; “urinary anastomosis”; “microsurgical anastomosis”; “anatomical variation”; “anatomical complexity”; “postoperative management”; “postoperative complications”; “rejection”; “long-term complications”; “renal replacement”; “replacement therapy”; “renal organoids”; “renal”; “artificial kidney”; “scaffolds”; “tissue engineering”; “bioprinting”; “organ-on-a-chip”; “xenotransplantation”. Abstracts of the resulting articles were then screened by all authors to select relevant articles for further use.

## Analysis of preoperative evaluation for kidney transplantation: pediatric and adult perspectives

The preoperative evaluation for kidney transplant candidates is a comprehensive, multidisciplinary process essential for ensuring safety and optimizing outcomes, yet its focus and structure differ markedly between adult and pediatric populations (Table 1).

**Table 1:** Differences between the management of adults and pediatric kidney transplantation candidates.

	Pediatric recipients	Adult recipients
Pre-transplant evaluation		
Timing Strategy	Strong emphasis on pre-emptive transplantation	Pre-emptive preferred but often limited
Cardiovascular Assessment	Limited unless risk factors	Extensive screening
Urological Assessment	Mandatory imaging; cystogram/urodynamics if needed	Selective unless prior urologic disease
Malignancy Screening	Rare but evaluated	Extensive; cancer-free interval may be required
Frailty Assessment	Not routine	Increasingly standard in older adults
Psychosocial Evaluation	Developmental and family-centered	Adherence capacity and social stability
Vascular Imaging	Selective; anatomical planning in small children	Frequent due to iliac calcification
Surgical technique		
Surgical Access	<15 kg transperitoneal; >15 kg retroperitoneal	Standard retroperitoneal iliac fossa
Vascular Anastomosis	Microsurgical; size mismatch common	Standard macrovascular technique
Vessel Selection	May require aortic/IVC anastomosis	External iliac artery/vein standard
Growth Adaptation	Consideration for vessel growth	Not required
Urinary Reconstruction	Tailored in CAKUT, augmentation, Mitrofanoff	Standard Lich-Gregoir

While both adhere to core principles outlined in guidelines such as Kidney Disease Improved Global outcomes (KDIGO), the pediatric evaluation is uniquely tailored to developmental stages, congenital conditions, and growth, whereas the adult assessment is heavily oriented toward managing accumulated comorbidities and age-specific risks (1). A dedicated multidisciplinary team is central to both pathways, but its composition and the weight given to specific assessments—such as neurocognitive development in children vs. frailty and cardiovascular disease in older adults—reflect these divergent priorities. In pediatric candidates, the evaluation is fundamentally growth- and development-centric. Critical components include a detailed urological assessment, particularly for children with congenital anomalies of the kidney and urinary tract (CAKUT), to ensure bladder suitability and plan potential surgical reconstruction (3). Psychosocial and neurocognitive screening is emphasized, especially for those who developed end-stage kidney disease at a young age, to address potential academic and developmental delays (1, 4). The immunization schedule must be verified and completed, and dental clearance is mandated to mitigate infection risk.

Pre-emptive transplantation is recommended as the preferred therapeutic approach for eligible patients, offering superior survival and quality of life compared to dialysis. As a timing strategy, it aims to perform transplantation prior to dialysis initiation; clinical benchmarks for this strategy in children include proceeding when the estimated glomerular filtration rate (eGFR) falls below 15 mL/min/1.73 m^2^ or earlier if symptoms of uremia develop (1, 5).

The decision to pursue pre-emptive transplantation hinges on the availability of a living donor and the timely completion of the candidate's evaluation, reinforcing the recommendation to begin the work-up 6 to 12 months before dialysis is anticipated (1, 5).

Conversely, the adult evaluation is dominated by comorbidity management and risk stratification. A thorough cardiovascular workup is paramount, given the high prevalence of atherosclerosis, hypertension, and diabetes in this population (6). Extensive screening for latent infections, malignancy, and psychosocial stability is conducted, with specific conditions potentially requiring treatment or a mandated cancer-free interval before listing (7). For older adults, the assessment incorporates geriatric-specific evaluations for frailty, sarcopenia, and cognitive decline, which are powerful predictors of surgical and post-transplant outcomes (8). The concept of prehabilitation—employing structured exercise, nutritional optimization, and psychological support—is increasingly integrated to enhance physiologic resilience before surgery. For the pre-transplant evaluation, the utility of routine vs. as-needed vascular imaging remains a topic of clinical discussion. A history suggesting previous iliac or femoral vein thrombosis should always initiate pre-operative imaging (such as CT with contrast, CT angiogram, or Doppler ultrasound) to establish patency of the iliac veins and the IVC, as an intra-operative finding of unexpected thrombosis may lead to abandonment of implantation (9). Similarly, many kidney transplant candidates are at risk for iliac arterial calcification. While a selective approach limits radiation and contrast exposure, proponents of routine, non-contrast CT imaging argue that it provides valuable three-dimensional anatomical mapping (10).

Several novel surgical approaches have been described to overcome these challenges. For instance, in patients with an absent or severely diseased common iliac artery, arterial reconstruction using the donor's iliac vessels can be performed to establish adequate inflow (11). More extensive arterial reconstruction with donor iliac vessels has also been successfully utilized in the setting of severe recipient atherosclerosis to facilitate transplantation (12). In cases of widespread recipient iliac disease, an alternative approach involves performing a recipient aortoiliac graft (e.g., using synthetic or biological material) to create a healthy vascular conduit for implantation (13).

Significant differences also exist in the management of specific risk factors. While both populations require detailed surgical planning, pediatric surgery frequently addresses technical challenges related to vessel size mismatch and prior abdominal surgeries, which are less common in adults. Immunological assessment is crucial for both groups but is often more complex in sensitized pediatric patients who may have undergone prior transplants or blood transfusions. While rarely a concern in pediatric candidates, a history of pre-transplant malignancy (PTM) is a major consideration in adults, associated with significantly higher post-transplant mortality and cancer recurrence, necessitating careful individualization of waiting periods and immunosuppression (14).

In summary, the preoperative transplant evaluation is a tailored, life-stage-specific process. The pediatric pathway prioritizes developmental integrity, congenital anomaly management, and fostering future growth, operating on a trajectory of lifelong graft function (15). The adult pathway, however, focuses on mitigating the risks imposed by chronic diseases, optimizing physiologic reserve, and navigating complex histories such as prior malignancy. Despite these differences, the overarching goal remains the same: a meticulous, patient centered on assessment that balances risks and benefits to ensure the best possible long-term survival and quality of life after transplantation.

## Precision and adaptation: the specific surgical techniques of pediatric renal transplantation

### Personalized surgical access and placement

The surgical strategy for pediatric kidney transplantation is meticulously tailored to the recipient's size. For children weighing less than 15 kilograms, a transperitoneal approach via a midline incision is typically employed to accommodate the adult-sized graft within the abdominal cavity (16, 17). For recipients over 15 kg, the kidney is usually placed retroperitoneally in the iliac fossa. In a significant innovation for the smallest infants under 5 kg, surgeons now utilize “super-minimal access” retroperitoneal techniques, which dramatically reduce tissue trauma and lower postoperative morbidity, facilitating a quicker recovery (2).

### The critical microsurgical anastomosis

The success of the transplant hinges on the vascular anastomosis—the delicate connection of the donor kidney's vessels to the child's circulation. This is a demanding microsurgical procedure performed under high magnification using surgical loupes or an operating microscope (18). To ensure precision and prevent clotting, the field is continuously irrigated with heparinized saline. The standard technique involves sewing the end of the donor renal artery and vein to the side of the recipient's external iliac artery and vein, respectively, using extremely fine, non-absorbable sutures (typically 7-0 or 8-0 Prolene) (16, 18, 19).

Although this suturing technique is regularly described in the literature, we prefer to perform vascular sutures in children with gradually absorbable Polydioxanone sutures (PDS). Venous sutures are performed with PDS 5-0 thread and arterial sutures with PDS 6-0 thread. The theoretical advantage of this approach lies in permitting progressive anastomotic expansion in parallel with somatic growth of the pediatric recipient. Such adaptive remodeling may decrease the long-term incidence of vascular stenosis or anastomotic stricture, which remain significant causes of graft dysfunction in growing children.

### Managing anatomical complexities

Surgeons must expertly adapt to anatomical variations in the donor kidney. A common challenge is the presence of multiple renal arteries. This is managed through advanced bench reconstruction techniques, where the arteries are surgically joined on a back table to create a single, common opening (a “Carrel patch”) for anastomosis, or alternatively, by performing separate, precise connections (18). The suturing must be continuous and entirely tension-free to avoid damaging the vessel lining, twisting, or narrowing, which could compromise immediate blood flow or lead to future stenosis.

### Advanced techniques for complex cases

For very young infants or children with unsuitable iliac vessels, an alternative, more advanced technique is utilized: direct aortic implantation. This involves a trans-peritoneal approach to access the child's aorta (3). A partial clamp is placed on this major artery, allowing the donor renal artery to be sewn directly onto its wall. While this method provides robust, high-volume blood flow to the graft, it requires exacting surgical skill to adjust the length of the vascular pedicle and prevent complications such as kinking, tension, or excessive blood flow to the new kidney (17).

Size mismatch between the graft and the recipient's abdominal cavity may result in increased intra-abdominal pressure, predisposing to abdominal compartment syndrome. This can compromise not only graft perfusion and function but also visceral organ perfusion and major vascular flow. Careful preoperative assessment of donor–recipient size matching, intraoperative monitoring of intra-abdominal pressure, and strategic planning of abdominal wall closure are essential.

### Urinary anastomosis technique

Prior to potential pediatric kidney transplantation, an ultrasound evaluation of the urinary tract must be performed. In cases of prior surgical history, urinary tract infections, or urinary incontinence, a cystogram should complement the evaluation. If bladder dysfunction is suspected, a urodynamic assessment (UDA) should also be conducted (20).

When the preoperative assessment is unremarkable, the urinary anastomosis is like that performed in the adult population. In most cases, a ureterovesical anastomosis is performed between the graft ureter and the bladder mucosa, using the Lich-Gregoir technique with either two 4-0 monofilament running sutures or interrupted sutures. As in adults, the ureter must first be trimmed as short as possible to avoid any risk of ischemic stenosis. It should also be spatulated to prevent anastomotic stricture. A stent is placed. An anti-reflux mechanism is then created to prevent vesicoureteral reflux. The bladder catheter is left in place for 5 to 7 days (21).

The specificities of urinary anastomosis in pediatric kidney transplantation lie in adapting the technique to the patient (weight, size) and the transplant context (22).

Although this suturing technique is regularly described in the literature for adult kidney transplantation, we prefer to perform urinary anastomosis with absorbable sutures PDS, using the Lich-Gregoir technique with either two 5-0 PDS monofilament running sutures or interrupted sutures. A JJ stent is placed. An anti-reflux mechanism is then created to prevent vesicoureteral reflux. The bladder catheter is left in place for 5 to 7 days.

### Specificities in CAKUT (congenital anomalies of the kidney and urinary tract)

Since CAKUT accounts for 40% of pediatric end-stage renal disease, pyelo-ureteral anastomoses using the patient's native ureter are generally contraindicated in this context (due to vesicoureteral reflux, megaureter, etc.) unless the preoperative assessment (cystography) is normal (22). In cases of a short ureter where a pyelo-ureteral anastomosis is not feasible, reimplantation onto a psoas-hitched bladder is performed. Given the complex and variable anatomy associated with CAKUT, close, real-time collaboration with a Pediatric Urologist in the operating room is crucial for intraoperative decision-making and to ensure the optimal surgical approach for these patients.

### Specificities in augmentation cystoplasty

In cases of augmentation cystoplasty, special care must be taken during exposure of the bladder and its augmentation patch to avoid compromising its vascular supply (23). The anastomosis is performed as postero-laterally as possible on the bladder to utilize the native bladder tissue for creating an anti-reflux mechanism (20). If this is not possible, the anastomosis is performed directly onto the bladder augmentation patch. In this scenario, the bladder catheter is retained for 10 days.

### Specificities in mitrofanoff or monti diversions

A Mitrofanoff-type diversion using the appendix or a Monti-type diversion using ileoplasty does not alter the preferred ureteral implantation site, which remains on the native bladder.

## Living donor kidney transplantation

Pediatric renal transplantation from a living donor stands as the definitive gold-standard treatment for children with end-stage renal disease. It offers a dramatically superior prognosis compared to long-term dialysis, providing exceptional benefits in patient survival, physical growth, and overall quality of life (24). This approach transforms a child's future, replacing the burdens of chronic dialysis with the potential for a (next-to) normal, active childhood. The ability to use a kidney from a living donor, most often a biologically related parent, is the cornerstone of this strategy, enabling a planned and optimized medical journey rather than an urgent response to organ failure (25).

The living donor pathway enables a critical advantage: pre-emptive transplantation. This means the surgery can be performed before the child ever requires dialysis, circumventing the developmental delays, nutritional deficits, and cardiovascular strain associated with chronic dialysis (26). The utilization of a living donor, most often a parent or close family member, is a particularly valuable strategy (24). Transplants from living related donors typically offer better Human Leukocyte Antigen (HLA) compatibility, which directly translates to a significantly reduced risk of acute rejection episodes (27). This biological advantage, combined with the elective nature of the surgery, contributes to shorter initial hospital stays, fewer complications, and a smoother postoperative recovery for the child.

The process begins with a comprehensive, voluntary evaluation of a healthy adult donor, ensuring their safety is the paramount concern. This elective framework allows for meticulous surgical planning and optimal preparation of the donor kidney. Comprehensive preoperative assessment of living kidney donors includes multiphase arteriovenous CT angiography with a dedicated urographic phase and high-resolution three-dimensional vascular reconstruction. This evaluation allows precise characterization of arterial multiplicity, venous anatomy, ureteral course, and potential anatomical variations critical for surgical planning. When both kidneys demonstrate acceptable vascular and urological anatomy, kidney selection for transperitoneal laparoscopic procurement is primarily guided by split renal function determined by radionuclide scintigraphy, preferentially preserving the kidney with superior function for the donor. Under controlled conditions, the donor nephrectomy is performed, often preferring the left kidney due to its longer renal vein, which facilitates the upcoming vascular connections in the recipient (28, 29). This careful selection and retrieval process guarantees the highest possible graft quality, a healthy, fully functional kidney delivered in an ideal state for implantation.

We have developed a laparoscopic nephrectomy protocol that allows for the harvesting of the graft from the related donor under maximum safety conditions, offering an increased prognosis of graft survival with the shortest possible warm and cold ischemia time (30).

Implanting an adult-sized kidney into a child's smaller abdominal cavity presents a unique surgical challenge. The placement and technique are meticulously adapted to the recipient's size. For very small children and infants, the kidney is often placed intraperitoneally through a midline incision to accommodate its size. For larger children and adolescents, a more standard retroperitoneal placement in the iliac fossa is used. The surgical approach, therefore, is not one-size-fits-all but is carefully personalized based on the child's weight and anatomy to ensure the graft fits without compression and functions properly from the moment of revascularization.

The culmination of this strategic pathway is a transplant with proven, superior long-term outcomes. Studies consistently demonstrate that pediatric living donor transplants have higher graft survival rates compared to those from deceased donors (31). The combination of a high-quality organ, better immunological match, pre-emptive timing, and precision surgery gives the child the best possible foundation for long-term health (32). Ultimately, a living donor kidney transplant is more than a procedure; it is a durable gift that offers a child the greatest chance at a full life, free from dialysis, with a functioning kidney that can grow with them into adulthood (25). The precision of the vascular anastomosis is ultimately determinant for ensuring immediate graft function and long-term durability, making it a technically demanding but optimally beneficial treatment for children.

## Outcomes in postoperative management of adult and pediatric kidney transplantation

Postoperative management following kidney transplantation is crucial for both short-term recovery and long-term graft survival, yet the priorities and predominant complications differ significantly between pediatric and adult recipients. The pediatric population's care is uniquely shaped by developmental trajectories, growth-related goals, and age-specific adherence crises (33). Adults face a greater burden of metabolic and cardiovascular comorbidities, making conditions like hypertension (incidence: 50%–90%) and post-transplant diabetes (10%–30%) central challenges (34, 35). While excellent one-year patient survival exceeds 95% in both groups, five-year graft survival rates highlight a disparity: pediatric recipients achieve approximately 85%–90% (up to 93% with living donors), whereas adult rates are generally lower, around 70%–80% for deceased donor grafts, largely influenced by competing mortality from cardiovascular disease (36, 37).

### Delayed graft function

The incidence of delayed graft function (DGF) in pediatric kidney transplant recipients is approximately 5%–6% for recent transplants, with higher rates observed in deceased donor transplants compared to living donor transplants (38). In the early postoperative period, adults contend more frequently with DGF, with a reported incidence of 30%–50% in deceased donor transplants, particularly from donors after circulatory death. Major risk factors include prolonged cold ischemia time (CIT), high donor serum creatinine, long pre-transplant dialysis duration, greater HLA mismatch, higher donor BMI, older donor age, and recipient diabetes (39). Prolonged CIT is especially significant, as each extra hour of cold storage increases ischemia– reperfusion injury risk. Deceased donor kidneys—particularly from donors after circulatory death—are more prone to DGF than living donor kidneys, with rates of 30%–50% (40). Recipient factors, such as diabetes and extended dialysis history, further heighten the risk, highlighting the need for careful donor selection and perioperative planning (39).

### Infections

Urinary tract infections (UTIs) are a frequent and serious problem after kidney transplantation, with the potential to harm graft function and patient survival. KDIGO guidelines recommend preventive measures such as trimethoprim–sulfamethoxazole (TMP-SMX) prophylaxis for at least six months and early removal of catheters or stents within four weeks to reduce risk (41). Asymptomatic bacteriuria is usually not treated unless persistent or linked to graft dysfunction, while symptomatic UTIs require targeted antibiotic therapy based on local resistance data. Complicated infections, including pyelonephritis, require hospitalization, broad-spectrum IV antibiotics, and imaging to rule out abscess or obstruction (42). Recurrent UTIs may need secondary prophylaxis like fosfomycin or assessment for anatomical abnormalities. For multidrug-resistant cases, emerging options such as fecal microbiota transplantation and bacteriophage therapy are being explored (43). Optimal management depends on coordinated care between infectious disease, nephrology, and urology teams.

### Acute rejection

The landscape of acute rejection presents differently across age groups. In pediatric recipients, the incidence of kidney graft rejection is approximately 10%–15% for acute rejection within the first-year post-transplant, with cumulative rates reaching 23% at 1 year and 39% at 5 years (2, 44). While young children have strong immunological responsiveness, non-adherence emerges as the single most potent risk factor for rejection and late graft loss in adolescents, contributing to this age group having the highest rates of graft failure (44, 45).

In adults, the incidence of biopsy-proven acute rejection in the first-year ranges from 10%–20%, with immunological risk driven by cumulative exposures. Key immunological risks include HLA mismatches, preformed donor-specific antibodies (DSAs), and high panel reactive antibody (PRA) levels, particularly in sensitized recipients (e.g., those with prior pregnancies, blood transfusions, or previous transplants) (46). ABO incompatibility also elevates rejection risk, though desensitization protocols can mitigate this (1), non-immunological factors include donor characteristics (e.g., older age, deceased donation, marginal donors with comorbidities like hypertension or diabetes), prolonged cold ischemia time (>15 h), and recipient factors such as as Black or African American genetic ancestry, younger age, and non-adherence to immunosuppressive therapy (47).

### Long-term complications

Long-term complications also reflect age-specific vulnerabilities. Pediatric recipients must navigate growth and development alongside long-term risks. Successful catch-up growth is a key metric, with the mean height-for-age Z-score improving from approximately −2.3 to −0.8 post-transplant (37, 48). The long-term clinical landscape is further complicated by non-immunological sequelae, including a notable risk of post-transplant lymphoproliferative disorder, particularly in EBV-naïve recipients, and the pervasive burden of CKD-mineral and bone disorder, which compromises bone integrity (49). Adults, on the other side, are predominantly affected by cardiometabolic disease. Post-transplant hypertension is nearly ubiquitous (70%–90%), and cardiovascular disease remains the leading cause of death (50).

In conclusion, the postoperative journey for pediatric recipients is a life-course model focused on supporting development and preventing adherence-related graft loss, while managing significant risks like PTLD, whereas adult kidney transplant recipients largely follow a chronic disease management model focused on mitigating high-incidence cardiometabolic risks to preserve the graft. The unifying principle is lifelong, multidisciplinary care, but the specific targets—from ensuring growth in children and preventing rejection in adolescents to managing prevalent hypertension in adults —must be precisely tailored to their distinct biological and psychosocial landscapes.

## Surgical complications

### Vascular and lymphatic complications

Vascular and lymphatic complications following kidney transplantation vary significantly between adult and pediatric populations in their incidence, risk profile, and clinical implications. Arterial complications primarily stenosis and thrombosis are relatively uncommon overall but carry distinct patterns. In children, these remain leading threats to graft viability, with renal artery thrombosis (RAT) being a predominant cause of early graft loss, particularly in young recipients, grafts from donors under six years, and en-bloc transplants (51). Technical challenges due to size mismatch and prior abdominal surgery elevate pediatric risk. In adults, the incidence is approximately 1%–3% for stenosis and 0.08%–1% for thrombosis. Renal artery stenosis (RAS) also shows a divergent risk profile: in children, end-to-end anastomosis to the internal iliac artery poses higher risk than end-to-side anastomosis to the iliac vessels. Additionally, less frequent arterial complications encompass anastomotic hemorrhage and, rarely, immune-mediated vasculitis (e.g., post-viral etiologies such as SARS-CoV-2), which may necessitate surgical or endovascular intervention (52). In contrast, in adults, atherosclerosis and clamp injury are common contributors (51, 53).

Venous complications, though rare overall (0.1%–1% incidence), are particularly consequential in pediatric recipients. Renal vein thrombosis (RVT) represents a critical early postoperative event in children, strongly associated with recipient weight <15 kg, young donor grafts, and technical anastomotic difficulty (51, 54). In adults, risk factors for these complications include patient-related factors (e.g., atherosclerosis, diabetes, hypercoagulability), surgical factors (e.g., clamp injury, anastomotic technique, donor-recipient vessel mismatch), and postoperative factors (e.g., CMV infection, immunosuppression, early mobilization) (55).

Post-biopsy vascular injuries, such as arteriovenous fistulas (AVFs) and pseudoaneurysms, occur in 0.2%–2% of cases and are often linked to larger needle gauges and inadequate monitoring in both populations (56). Lymphatic complications, notably lymphoceles, occur more frequently, with reported rates of 0.6%–22% across all ages. In adults, risk is heightened by immunosuppression, surgical technique, and early mobilization; in pediatric patients, prior surgeries and size disparities also contribute (56).

Management strategies remain broadly aligned, though tailored to patient size and anatomy. Arterial stenosis is typically treated with endovascular angioplasty and stenting in both groups, while arterial thrombosis may require thrombolysis or thrombectomy (57). Venous thrombosis demands urgent intervention mechanical thrombectomy or anticoagulation though salvage is often challenging, especially in pediatric cases where graft loss is rapid (58).

Lymphoceles are initially managed with percutaneous drainage, with sclerotherapy or surgical fenestration reserved for refractory cases (59). Biopsy-related complications are commonly addressed with transcatheter embolization.

Early detection via Doppler ultrasound and cross-sectional imaging is critical across all age groups (56). Minimally invasive endovascular approaches are preferred when feasible, though pediatric cases may necessitate specialized techniques due to vessel size. Prophylaxis through meticulous surgical technique, careful donor-recipient matching, and vigilant postoperative monitoring is essential in both populations to mitigate these risks and optimize long-term graft survival.

### Urological complications

The incidence of urological complications following kidney transplantation (KT) ranges from 2.9% to 20%, with ureteral stenosis, urinary leaks, and vesicoureteral reflux (VUR) being the most common issues (60). These complications often arise within the first three months post-transplant and can significantly impact graft function and patient morbidity. Ureteral stenosis, occurring in 2%–10% of cases, is frequently caused by ischemia or surgical technique, while urinary leaks (up to 10%) typically result from anastomotic insufficiency. VUR, with an incidence varying from 10.5% to 86%, is more prevalent in deceased donor grafts and may contribute to recurrent urinary tract infections (UTIs) or graft dysfunction (60). Early diagnosis and intervention are critical to preserving graft function and minimizing long-term adverse outcomes.

Minimally invasive techniques have become the cornerstone of managing urological complications post-KT, offering reduced surgical risk compared to open reintervention. Endoscopic approaches, such as balloon dilation, laser incision, or stent placement, are effective for ureteral stenosis, with success rates ranging from 57% to 88% (61). For urinary leaks, initial management often involves percutaneous nephrostomy or ureteral stenting, with surgical revision reserved for refractory cases. VUR can be managed conservatively with antibiotics or treated endoscopically using bulking agents, which have shown success in reducing UTIs (60, 61). Additionally, stone disease, though rare (1% incidence), is managed similarly to native kidneys, with extracorporeal shockwave lithotripsy (SWL) or ureteroscopy being preferred (61). These strategies highlight the importance of tailored, minimally invasive interventions to optimize outcomes in KT recipients.

### Diagnosis of early post-transplant surgical complications

Accurate diagnosis of perigraft fluid collections following kidney transplantation is essential for appropriate management, as urinomas, lymphoceles, hematomas, and abscesses require distinct therapeutic approaches. (Table 2) Doppler ultrasound serves as the initial imaging modality of choice, enabling visualization of fluid collections and assessment of their relationship to the graft (56). However, imaging characteristics alone may be insufficient for definitive diagnosis, necessitating biochemical analysis of aspirated or drained fluid. For urinomas, fluid analysis reveals creatinine levels significantly higher than serum creatinine due to direct communication with the urinary collecting system (60). Lymphoceles are characterized by fluid with creatinine and electrolyte levels like serum, elevated lymphocyte count, and triglyceride-rich content reflecting lymphatic origin (59). Hematomas typically present with hematic fluid and may show declining hemoglobin levels, while abscesses demonstrate purulent appearance with positive cultures and elevated neutrophil counts. Cross-sectional imaging with CT or MRI provides complementary information regarding collection location, loculation, and relationship to adjacent structures, with CT lymphangiography offering definitive diagnosis for refractory lymphatic leaks through direct visualization of contrast extravasation (56).

**Table 2:** Immediate surgical complications.

Fluid collection type	Fluid analysis	Typical timing post-transplant	Key distinguishing features
Urinoma	Creatinine >> serum creatinine (often >10×); potassium like urine; cell count variable	Early (first days to 3 weeks)	Direct communication with collecting system; fills with contrast on delayed imaging or nuclear scintigraphy
Lymphocele	Creatinine like serum; triglycerides elevated (>100 mg/dL); cholesterol variable; lymphocyte count >90%; sterile	4–8 weeks (peak ∼6 weeks)	Triglyceride-rich fluid; no epithelial lining (pseudomembrane); may cause mass effect on graft or ureter
Hematoma	Bloody aspirate; hemoglobin present; declining serial hemoglobin levels (>20 g/L drop over 24 h); sterile unless infected	Acute (within days of surgery or biopsy)	Associated hemoglobin drop; sterile unless secondarily infected

## The future in pediatric transplantation

As in adult surgery, the trend is toward minimally invasive surgery. The first robot-assisted transplant was recently described in 2019 (62). Subsequent studies have demonstrated the safety and reproducibility of the procedure, provided it is performed in an expert center. Decaestacker's team recently clearly identified the challenges of robotic surgery in pediatrics: the use of instruments designed for adults, a restricted abdominal cavity with reduced working space, and the lack of a standardized technique for trocar placement due to significant disparities in patient weight and size, necessitating individual adjustment for each case (63). Therefore, pediatric robot-assisted transplantation currently appears demanding, and the associated learning curve confines this technique to high-volume centers. At present, this technique is not recommended for routine use.

## Alternative methods to replace renal function emerging from the field of bioengineering

Four types of precursor cells are required for the creation of a kidney, namely nephron progenitor cells ultimately forming nephrons (consisting of glomerular podocytes, Bowman's capsule, Henle's loops and renal tubules); ureteric bud cells forming the collecting duct and ureter, stromal progenitor cells that form the renal interstitium; and vascular progenitor cells differentiating into the vascular renal network (64). The saga began with the establishment (and *in vitro* expansion) of pluripotent mouse stem cells in the early 1980s, allowing besides genetic studies—their main initial purpose—the origination of all germ layers and with that, virtually every type of mammalian tissue (65, 66). It was not until 1998 that the derivation and expansion of human embryonic stem cells was established. However, ethical issues surrounding the use of human embryos hindered swift development (67). Since the dawn of human induced pluripotent stem cells (hiPSCs) in 2007 the scene changed drastically as human embryos were no longer required for their derivation (68, 69). Following this development, the induction and isolation of human renal cell lineages thrived (70, 71). More recent developments such as the advent of the CRISPR/Cas9 gene-editing method has further advanced the field by enabling rapid genetical engineering of hiPSCs (72).

### Renal organoids

Different kinds of renal organoids have since been developed from hiPSCs, notably nephron type organoids and ureteric bud/ collecting duct type organoids, but not yet stromal type organoids, and although endothelial cells are present in these organoids, structural vascularization is largely lacking as well (64, 73). Organoids composed of all anticipated kidney cell types with separate glomerulus, proximal and distal tubule, renal interstitium and an extensive endothelial network have thus far been created from mouse embryonic stem cells only, and are useful for developmental understanding, modelling of kidney diseases, drug/toxicology screening and tissue engineering, but not for transplantation (74).

The state of the art of available *in vitro* organoids does not allow the creation of a human kidney size 3D organ. The main challenges in organoid development are developmental complexity in relation to function, maturity and scale in relation to structure.

Structures *in vitro* form simultaneously departing from the evolutionary consecutive way of development of the human kidney: the ureteric bud arises at week five post conception while nephrogenesis continues until week 36 (64). These differences result for example in nephrons and collecting ducts that are not integrated correctly in the organoids. Furthermore, not all elements originating from the nephron and the ureteric bud are available as organoids. Existing human cell-derived organoids do not have a functional ureter and currently available nephron type organoids produce mostly glomeruli and proximal renal tubules, not distal renal tubules and loops of Henle (64, 71, 72, 75). Improved differentiation strategies are needed to get to a basic comprehensive level.

Pure organoids generated with currently available protocols achieve a maturation rate equivalent to an embryonal stage corresponding to the first trimester of human gestation (76). Further maturation of all renal segments available as organoids is needed. It is not yet clear if maturation needs to be complete *in vitro* or can be partially *in vivo* after transplantation (64). In the process of improving maturity, off target populations and contamination with undifferentiated PSCs need to be eliminated (72, 77).

The lack of vasculature in current organoids means that oxygen, nutrients and other elements are not transported efficiently across the structure, and that “renal” function is partial at best (64, 76). When hiPSC-derived podocytes were transplanted under the kidney capsule of a mouse, maturation towards vascularized glomeruli connecting to host-derived vessels was possible (78). This suggests that the connection to tissues present in the host theoretically may be able to overcome certain issues that are linked to the *in vitro* environment.

### Organoids and scaffolding

To overcome the issues with structure, seeding (recellularization) of decellularized biological or hybrid (electrospun) scaffolds or seeding of purely synthetic scaffolds with functional parenchymal cells of engineered progenitor cells is attempted (79, 80). So far however, orthotopic transplantation of these scaffolds into nude mice led to low urine output and subnormal levels of renal function (i.e., albumin retention, reabsorption of electrolytes and glucose) (81). Allocating the various cell types to their appropriate positions proves highly challenging (73). In the respiratory tract, decellularized—recellularized scaffold tissue was successfully used to treat acute bronchial disease in a human patient in 2008; autologous stem cells were used to recellularize the graft and no immunosuppressants were necessary for graft survival (82).

### 3D bioprinting

In 3D bioprinting, hydrogels containing cells are deposited in a precise layer-by-layer manner to generate complex tissue or organs (83). This has been successfully applied to create cartilage, skin, nerves and cardiac tissues for use in reconstructive surgery (84). The structure of the human kidney has proven too complex thus far with its arteries, veins, ureter, and approximately one million functional nephron units and stroma in between, its many required cell types (∼20 types in the mature kidney) and its demanding standards complicating the choice of biomaterial to create a sustainable organ (4, 26, 85, 86). Portions of the nephron have been successfully generated using 3D bioprinting techniques and the combination of 3D bioprinting with organoids may be promising if it keeps evolving (87–90).

### Organ-on-a-chip

This technology reproducing human organ architecture on a miniature scale was originally developed to model disease and perform drug tests; since its emergence several functions of the kidney have become available, such as “glomerulus-on-a-chip” and “tubule-on-a-chip”, however combining all kidney functions on a chip does not (yet) seem possible (91, 92). Combining chips and kidney organoids resulted in improved vascularization and maturation of podocytes and renal tubules, however significant filtration and an excretion route are still lacking (93).

### Wearable artificial kidneys

The goal of these miniature devices is to allow patients to be dialyzed whilst performing their usual activities (94). They consist of sensors, pumps, membranes and filters that clean and re-use dialysate solutions without the use of large quantities of water. However, thus far pump dysfunction, bubbles and clotting in the circuit and battery failure, have hampered clinical application (95).

Filters and human kidney cells are combined in these surgically positioned devices, that are in development but not yet on the market, mainly due to issues regarding human cell selection (95, 96).

### Interspecies blastocyst complementation

The technique of injection of (h)iPSCs into an animal that is genetically modified to lack the kidney with the goal of replacing the lacking organ led to the originating of a functional nephron connecting to the native ureter and bladder in a deficient rat receiving mouse embryonic stem cells (97). For obvious reasons, extreme caution needs to exercise when creating such chimeras between humans and animals.

### Interspecies progenitor replacement

Injection of PSCs during renal development stage rather than blastocyst stage may avoid allocation of injected cells off-target and has been successful when mouse or rat nephron progenitor cells were injected into genetically manipulated mice, but did not function efficiently when hiSPCs were injected, potentially because of the large evolutionary distance between these species (98, 99).

### Xenotransplantation

Parallel with the efforts in creating organoids (combined with compatible technologies), a combination of genome editing and xenotransplantation is applied to overcome the shortage of organ donors. Recently, the first genetically-modified-pig-to-human cardiac transplant was performed—the patient survived 60 days before succumbing to infectious complications (100). Genetically-modified-pig-to-human kidney transplant has been performed in brain dead patients only and final evaluation was at three days post-transplant (101). Considering this short time frame, results were mildly encouraging with routine immunosuppression only, no hyperacute rejection was observed and no porcine viruses were detected. Furthermore, the higher human baseline blood pression did not lead to vascular damage at this short term. Urine was excreted but serum creatinine did not decrease. Combining replacement of renal progenitor cells by hiPSC-derived cells into genetically modified pigs may provide a promising route to escape the need for immune suppression (86).

Soon, a combination of the many methods and techniques described above, will likely lead to a viable alternative to human-to-human kidney transplantation and as such help diminish the ratio between the number of patients and available organs.

## Conclusion

In conclusion, the journey of kidney transplantation is a testament to precision medicine, demanding distinct yet equally rigorous pathways for pediatric and adult recipients. While the goal of achieving long-term graft function and improved quality of life remains universal, the routes diverge significantly: pediatric care is fundamentally shaped by a developmental trajectory, prioritizing growth, congenital anomaly management, and navigating adherence crises, whereas adult care is dominated by mitigating the burdens of chronic comorbidities and age-related physiological decline. This comparative analysis underscores that excellence in transplantation is not a one-size-fits-all endeavor but is achieved through life-stage-specific adaptations from tailored evaluation and surgical technique to complication management and long-term surveillance. Embracing these specialized paradigms within a dedicated multidisciplinary framework is essential to ensuring the best possible outcomes across the entire lifespan of every transplant recipient. Meanwhile, hard work from bioengineers may enable alternative methods to replace kidney function in the decades to come.
